# Adverse Effects of Androgen Deprivation Therapy for Prostate Cancer: Prevention and Management

**DOI:** 10.1155/2013/240108

**Published:** 2013-07-25

**Authors:** Petros Sountoulides, Thomas Rountos

**Affiliations:** Urology Department, General Hospital of Veria, 55133 Thessaloniki, Greece

## Abstract

The prostate is an androgen-dependent organ. The increase, growth, homeostasis, and function of the prostate largely depend upon the intraprostatic and serum concentrations of androgens. Therefore, androgens are essential for the physiologic growth of prostatic epithelium. Prostate cancer, the second leading cause of death for men, is also androgen dependent, and androgen suppression is the mainstay of treatment for advanced and metastatic disease. In the state of metastatic disease, androgen suppression is a palliative treatment leading to a median progression-free survival of 18–20 months and an overall survival of 24–36 months. Theoretically, the majority of patients will develop hormone-refractory disease provided that they will not die from other causes. Although androgen suppression therapy may be associated with significant and sometimes durable responses, it is not considered a cure, and its potential efficacy is further limited by an array of significant and bothersome adverse effects caused by the suppression of androgens. These effects have potentially significant consequences on a variety of parameters of everyday living and may further decrease health-related quality of life. This review focuses on the aetiology of these adverse effects and provides information on their prevention and management.

## 1. Introduction 

It is estimated that there are nearly 2.8 million men living with a history of prostate cancer in the USA, and an additional 241,740 cases will be diagnosed in 2012 [1]. Androgen deprivation therapy (ADT) is undoubtedly the mainstay of treatment for symptomatic metastatic prostate cancer. Although ADT indications are limited to the palliation of symptomatic metastases, ADT is widely used in men with biochemical (PSA) relapse after radical prostatectomy, locally advanced disease, lymph node metastases, and also asymptomatic metastatic disease [2, 3]. ADT is also commonly used in combination with external beam radiotherapy (EBRT) for intermediate to high-risk prostate cancer cases in order to improve responses to radiotherapy [4]. In total, it is estimated that approximately 40% of patients diagnosed with prostate cancer will receive ADT within 6 months of diagnosis [5].

Although there is no doubt that ADT is efficacious in delaying disease progression and alleviating symptoms from metastatic disease, ADT is associated with multiple and significant side effects. Given this, it is considerate to assign patients to ADT only when this is necessary and delay its implementation in order to spare the patients some of the associated morbidity associated with androgen withdrawal. However, studies have demonstrated that early versus deferred initiation of ADT is beneficial for patients with advanced disease. Significant survival benefit of early hormonal therapy has been observed among patients with asymptomatic metastatic disease, node-positive but clinically localized disease after radical prostatectomy and lymphadenectomy, and advanced local/regional disease during and after radiotherapy [6]. Additional evidence in support of early treatment initiation was provided by the Medical Research Council study of 938 patients with locally advanced or asymptomatic metastatic prostate cancer. Patients received either immediate treatment with orchiectomy or LHRH agonist versus the same treatment deferred until symptoms occurred. Development of extraskeletal metastases, pathologic bone fractures, spinal cord compression, and ureteric obstruction was twice as common in the deferred treatment group. Overall survival was significantly prolonged in the patients who were treated early [7].

Given the current role of ADT and its expanding indications in prostate cancer treatment, concerns have been raised relevant to the documented side effects of this treatment and its overall effect on quality of life (QoL). ADT is accompanied by an array of side effects and toxicities. And while sexual side effects including loss of libido and erectile dysfunction are well recognized and anticipated, changes in body composition (gynecomastia, weight gain, reduced muscle mass and muscle tone, and increase in abdominal fat), cognitive defects (memory loss) and metabolic disturbances (hyperglycemia, altered lipoprotein profile, decreased insulin sensitivity, and osteoporosis) are less commonly recognized side effects of ADT. Additionally, both the diagnosis of prostate cancer and ADT itself can negatively affect psychosocial well-being and cause distress. Physicians should be aware of far-reaching consequences of ADT and should incorporate strategies for preventing and managing toxicities into routine practice [8].

## 2. The “Flare” Phenomenon

LHRH agonists are well known to cause a surge in serum testosterone levels during the first week of therapy due to the initial stimulation of LHRH receptors, the so-called “flare” phenomenon. The flare phenomenon was considered to be the cause of significant sequela if LHRH agonists are administered to men with high-volume metastatic disease. 

However, there are wide discrepancies regarding the frequency and severity of acute clinical progression that might result from the testosterone surge. The clinical consequences of the flare phenomenon are considered to be prevented by the concomitant administration of antiandrogens. Antiandrogens inhibit the stimulatory effect of the testosterone surge at the level of the androgen receptor, although there is not a clear consensus as to whether antiandrogens should be routinely given to all patients during the first month of LHRH therapy to prevent flare responses [9].

A recent study on 1566 metastatic prostate cancer patients treated with LHRH agonists showed that antiandrogen therapy before LHRH agonists was not associated with differences in fractures, spinal cord compression, bladder outlet obstruction, or narcotic prescriptions. Rates of spinal cord compression or fracture were <1% in the first 30 days after beginning LHRH agonist therapy regardless of antiandrogen use [10].

## 3. Hot Flashes

The symptom of hot flashes is among the most common and early described side effects of ADT as it was reported by Huggins and Hodges in 1941 in 9 of the first 21 prostate cancer patients ever to undergo ADT. Hot flashes are caused by inappropriate stimulation of thermoregulatory centers in the hypothalamus, resulting in peripheral vasodilatation [11]. Hot flushes are described by patients as the perception of intense warmth and subsequent cooling, flushing of the skin, perspiration, and chills in the upper part of the body, usually the neck and face. Associated symptoms may include anxiety and palpitations. Hot flashes usually last from a few seconds to several minutes but can persist for up to 20 min. Many patients only report mild consequences from their symptoms and only experience these sporadically. However, some patients experience multiple hot flashes each day and report significant effect on daily functioning and quality of life [12].

Relevant to the aetiology of this phenomenon, hot flashes in men under ADT develop due to the acute withdrawal of sex hormones, similar to what happens in women at the onset of menopause. It has been speculated that androgen withdrawal disrupts the equilibrium of the neurotransmitters increasing the levels of neurotransmitters, norepinephrine and serotonin, and hormones, including testosterone. This effect in turn is postulated to deregulate the homeostatic mechanism of the thermoregulatory centers in the preoptic zone of the hypothalamic [13].

The incidence of hot flashes, which may not abate over the course of ADT, is close to 80%. However, the incidence of hot flashes seems substantial even in men who are not under ADT or surgical castration. Results of a survey showed that a third of uncastrated men aged 55–75 years experienced hot flashes, of whom 15% had bothersome hot flashes [14]. 

Treatment options for hot flashes include a variety of options ranging from estrogens to antidepressants, anticonvulsant agents, and even acupuncture. According to a recent review, diethylstilbestrol, megestrol acetate, and cyproterone acetate provide the greatest efficacy, up to a greater than 75% decrease of the number of hot flushes, although this improvement comes with the risk of bothersome side effects [15]. However, since cyproterone is a drug approved for the treatment of prostate cancer, its use could interfere with hormonal therapy, and medroxyprogesterone could be considered the standard treatment for hot flushes in men undergoing androgen suppression for prostate cancer [16].

Estrogens, in particular DES (0.5–1 mg/day), has been effective in alleviating hot flashes in 75–90% of men, although concerns about the safety of these agents were raised given the high incidence of painful gynecomastia [17]. 

Megestrol acetate, a progesterone derivative, achieved an 85% reduction in hot flashes, compared to a 21% reduction in patients under placebo. A 2-3 week course of therapy is required to obtain maximal symptomatic reduction, with symptomatic relief lasting for several weeks after therapy [18]. Initial enthusiasm for megestrol acetate has been somewhat tempered by reports of elevations in serum PSA levels with subsequent decline in PSA levels upon its withdrawal [19, 20]. Therefore, although the drug is effective, PSA levels should be closely followed while on treatment. 

Newer antidepressants, particularly selective serotonin reuptake inhibitors (SSRIs-paroxetine 10 mg/day) and serotonin-norepinephrine reuptake inhibitors (SNRIs-venlafaxine 37.5 mg/day), are thought to alleviate hot flashes by increasing serotonin levels and by altering the neurotransmitter milieu within the thermoregulatory center [21]. Therefore, there is a reason to believe that they might also reduce the frequency and severity of flushing in men with prostate cancer under ADT [22]. A moderate effect of both SSRIs/SNRIs on hot flushes is to be anticipated, however, no results from RCTs are available [15, 16].

Gabapentin, a gamma-aminobutyric acid analogue, was originally developed for the treatment of epilepsy and neuropathic pain. However, it proved efficacious in controlling hot flashes in women with breast cancer, and for that reason it was also tested in men under ADT. Recent evidence on long-term treatment showed that the effects of gabapentin were dose-dependent leading to a moderate decrease in the frequency of hot flashes with moderate side effects [23, 24].

## 4. Sexual Dysfunction

ADT induces changes in serum testosterone that can result in changes in both sexual desire and function. The overwhelming majority of men under ADT will develop variable degrees of erectile dysfunction due to the lack of androgens. The two most important predictive factors for ED following ADT were age >70 and presence of diabetes mellitus [25]. Undoubtedly, ED can significantly affect the self-esteem, self-perception, and quality of life of younger, sexually active men, especially when coupled with the side effects of ADT on muscle and fat distribution [26]. Erectile dysfunction can be treated with both pharmacological agents and mechanical devices including phosphodiesterase inhibitors, vacuum devices, or penile injections of vasoactive agents. Response rates for pharmacotherapy range from moderate to good. 

Although these pharmacologic and mechanical approaches may restore the ability to achieve an erection, the additional loss of libido as a result of treatment often limits patients' motivation for pursuing treatment to restore erections [27]. Lately, the cautious use of estrogens has also been proposed for the improvement of both sexual interest and ED in these men [28].

## 5. Skeletal Morbidity 

Skeletal morbidity in the form of bone metastases, bone pain, osteoporosis, and associated fragility fractures is a burden to men with advanced and metastatic prostate cancer under ADT. It is a known fact that bone turnover and development are androgen mediated. Androgen deprivation causes a 3–5% annual decrease in bone mineral density (BMD) and prostate cancer patients on ADT have a 6–17% lower BMD than eugonadal men with prostate cancer [29]. 

There is an estimated up to sixfold increase per year in the risk of fractures for men under ADT compared to controls due to the ADT-induced osteoporosis [30, 31]. The fracture risk cumulatively increases with the duration of ADT starting from 5% at 5 years after initiation of ADT and reaching 20% at 10 years after initiation of ADT [32, 33]. Surprisingly, the overall fracture risk is also high in men with prostate cancer not treated with ADT; the estimated relative risk (95% CI) comparing men with prostate cancer to population controls was 1.35 (1.26–1.44) for femoral neck fractures and 1.33 (1.23–1.44) for intertrochanteric fractures [34]. 

In any case a fracture is a landmark event in the life of men with prostate cancer under ADT. Hip fractures in men over the age of 75 for any cause carry a mortality rate of 30%, while bone fractures in patients with prostate cancer have been associated with adverse overall survival outcomes [35].

Therefore, androgen ablation is a cause of skeletal-related events associated with significant morbidity and mortality even for patients with nonmetastatic prostate cancer. Risk factors for osteoporotic fracture include the duration of ADT (>3 years), age (mainly through decreased testosterone levels), ethnicity (Caucasian patients are at greater risk), smoking, lower BMI, and medications (e.g., glucocorticoids) [36, 37].

### 5.1. Treatment to Reduce Bone Loss and Skeletal-Related Events

Lifestyle modifications that apply to all men under ADT irrespective of their bone status include regular light weight lifting or resistance exercises, cessation of smoking, limiting alcohol and caffeine consumption, and vitamin D and calcium supplementation [29].

The management of ADT-induced osteopenia and osteoporosis has been a field of significant evolution during the last years, as bone-targeted therapies have recently been the focus of considerable research and drug development. The osteoclast has been recognized as a validated therapeutic target in the management of prostate cancer. Osteoclast inhibition with bisphosphonates reduces the risk for skeletal events in men with castration-resistant prostate cancer metastatic to bone. Osteoclast activity inhibition improves bone mineral density, a surrogate for osteoporotic fracture risk. Late generation bisphosphonates such as zoledronic acid (Zometa, Novartis Technology) seem to markedly reduce bone resorption and increase BMD in prostate cancer patients under ADT [38]. Zoledronic acid has also shown efficacy in preventing bone metastases and skeletal-related events in patients under ADT reducing by 7-fold the risk of pathological fractures after 20 months of treatment [39]. Certain precautions should be taken with the use of zoledronic acid; the risk of osteonecrosis of the jaw is further limited if patients are asked to refrain from dental procedures while on therapy, while the dose and administration of the drug (3 mL intravenous infusion in no less than 15 minutes) should be adjusted for patients on CKD.

As far as prevention strategies are concerned, BMD screening of all men on ADT at baseline and further annually or biennially evaluations have been recommended by some authors although routine BMD testing is not the rule [33, 35, 37]. There is no doubt that BMD should be closely monitored in patients under ADT, as data suggest that a low BMD (*T*-score > 2.5, or >1 in conjunction with other risk factors) before ADT initiation indicates a high risk of subsequent nonmetastatic fractures. BMD *T*-scores of >2.5 should call for immediate initiation of treatment. 

Other hormonal therapies tested for their potential benefits in bone protection include estrogen therapy using agents such as diethylstilbestrol. Estrogens have been shown to reduce the risk of osteoporosis onset as effectively as bisphosphonates, however, with the constant risk of cardiovascular adverse events. Studies with estrogen receptor modulators have also yielded promising preliminary results [40]. 

Currently, however, zoledronic acid (Zometa, Novartis Oncology), a newer generation bisphosphonate, and denosumab (XGEVA, Amgen) are the only bone-targeted therapies that have provided solid evidence of reducing the risk for skeletal events (SREs) among men with bone metastases and a rising PSA level despite a testosterone level <50 ng/dL (castration-resistant prostate cancer (CRPC)). 

Denosumab (XGEVA, Amgen) has recently received US Food and Drug Administration (FDA) approval to prevent skeletal-related events in cancer patients with solid tumors and bone metastases. The same drug marketed in certain European countries under the trade name Prolia is currently approved for the management of osteoporosis. Denosumab is a fully human monoclonal antibody that targets receptor activator for nuclear factor *κ*B ligand (RANKL). RANKL is a key mediator of osteoclast activity and bone destruction; therefore, its neutralization suppresses osteoclast activity.

The time to first bone metastasis and risk for symptomatic bone metastasis were significantly better with denosumab treatment compared to placebo as has been shown in a phase III trial that enrolled 1,432 men with CRPC, no bone metastases, and at least one feature consistent with a high risk for the development of bone metastases (PSA ≥ 8 ng/mL or PSA doubling time ≤ 10 months) [41]. Denosumab was significantly more efficacious than zoledronic acid at reducing skeletal-related events in a phase III study with 1904 castration-resistant prostate cancer patients in 39 countries [42]. 

Results from the same group have also shown denosumab to be effective in delaying bone metastasis in men with nonmetastatic castration-resistant prostate cancer. Denosumab significantly increased bone metastasis-free survival by a median of 4.2 months and also significantly delayed time to first bone metastasis compared with placebo without, however, any effect on overall survival [43]. Denosumab increases lumbar spine, hip, and radius bone mass density and reduces the risk of vertebral fractures in men receiving ADT for nonmetastatic prostate cancer [44].

Other potential advantages of denosumab include subcutaneous administration. The adverse effect profile is also different. There are no concerns about renal safety with denosumab, so there are no requirements for renal monitoring, which is a key requirement for bisphosphonate treatment.

 Beta-emitting radiopharmaceuticals are promising in reducing pain due to metastatic disease. Ongoing clinical trials involve alpha-emitting radium-223, the endothelin-A receptor antagonists atrasentan and zibotentan, and proto-oncogene tyrosine-protein kinase (SRC) inhibitor dasatinib [45]. Clearly, the evaluation that is underway of the modification of signaling proteins and cytokines that lead to the development and progression of androgen-independent cancer of the prostate (AICP) is important and will be a focus for several years to come.

## 6. Anaemia 

The association between androgens and erythropoiesis has been known for several decades. Androgens stimulate the hematopoietic system via mechanisms that include stimulation of erythropoietin release, increasing bone marrow activity and iron incorporation into the red cells [46]. Men with advanced or metastatic prostate cancer are prone to develop anemia due several causes. Anemia can be caused by either blood loss due to direct infiltration of the bladder or replacement of the bone marrow from metastatic disease. Moreover testosterone increases the production of erythrogenesis-stimulating proteins. Therefore, ADT in the form of LHRH agonists may cause or exacerbate anemia by indirectly inhibiting erythropoiesis. Androgen deprivation therapy, either in the form of nonsteroidal antiandrogens (NSAAs) or by LHRH-agonists and surgical castration, is associated with a >10% decline in haemoglobin levels in most men with prostate cancer, and symptomatic anaemia in *≈*10% of patients. Two-year androgen suppression resulted in a statistically significant decline of Hb, which had, however, no clinically apparent adverse effect on the three quality of life domains [47]. 

Studies have shown that s.c. administration of recombinant human erythropoietin (150 U/kg three times weekly) is an effective treatment for managing poorly tolerated anaemia in men with androgen-independent prostate cancer, achieving a greater than 10% increase in haemoglobin concentrations [48]. 

## 7. Psychological and Cognitive Effects

Hormonal therapy has also been shown to cause neurologic impairment, manifested by decreased cognitive function, mood, and self-esteem while also negatively effecting memory and attention [49]. Low levels of testosterone are significantly associated with depression in elderly men and testosterone replacement appears to reduce depressive symptoms in such patients. Consequently, depression seems to be common in men with prostate cancer [50]. Cognitive side effects are apparent almost immediately, as shown in a recent 3-month neoadjuvant trial and appear to be reversible after completing treatment; however, sometimes the effects are only partly reversible at 1 year [51]. On the other hand, recently a small study of 18 patients receiving 12 months of androgen suppression revealed preservation of global cognitive performances and failed to observe impairment of cognitive function [52]. 

High-dose estrogens can also be used to reduce the cognitive effects of androgen ablation, but the benefits of this should be balanced against the well-recognized risk of cardiovascular events [53]. 

There is evidence of an increased occurrence of depression, anxiety, low self-body image perception, sleep disturbances, and diminished quality of life in prostate cancer patients undergoing adjuvant androgen deprivation therapy (ADT) [54]. A combined resistance/aerobic exercise programme may lead to significant improvement in fatigue and cognitive function [44].

Although depression has been reported after a diagnosis of PC, whether ADT leads to or worsens depression is not clear. A study on 257 patients with nonmetastatic CaP receiving ADT for 1 year showed no association between worsening of depressive symptoms among nondepressed or depressed patients with nonmetastatic prostate cancer [55]. Although depression associated with ADT typically does not respond to antidepressants, these are commonly prescribed to prostate cancer patients.

## 8. Metabolic Syndrome and Cardiovascular Morbidity

Metabolic alterations caused by testosterone suppression may mediate the mechanisms underlying the high frequency of cardiovascular disease that has been observed in some men under ADT. Concerns have been raised about the well-being of, particularly older, men on AST as there is evidence that ADT can lead to a symptom complex consistent with the metabolic syndrome. This syndrome is associated with an increased risk of death as a result of myocardial infarction, even in the absence of known cardiovascular disease or diabetes [56]. 

Some of these androgen deprivation therapy-related metabolic changes (obesity, insulin resistance, and increased triglycerides) overlap with features of the metabolic syndrome. However, in contrast to the metabolic syndrome, androgen deprivation therapy increases subcutaneous fat and high density lipoprotein cholesterol [57]. Toremifene has been shown to improve the lipid profile, while metformin coupled with lifestyle interventions is a safe treatment option for adverse metabolic changes [44].

Clinical evidence from two studies lend support to this association between ADT and cardiovascular morbidity [58, 59]. The increased cardiovascular toxicity was hypothesized to be mediated through changes in lipoproteins, arterial stiffness, and QT interval prolongation [60].

A recent study has corroborated previous findings suggesting that the use of ADT is associated with earlier onset of fatal MIs in men aged 65 years or older who are treated for 6 months compared with men who are not treated with ADT [61]. 

ADT has also been found to be the cause of decreased muscle strength due to its catabolic effect. Muscle weakness and impaired cognitive function are associated with an earlier decrease in functional capacity of the individual, compromising independent living and consequently decreasing quality of life [62]. Daily physical exercise is considered the key lifestyle modification in avoidance of these consequences of ADT as has been shown in relevant studies [63, 64]. 

## 9. Minimizing Androgen Deprivation Side Effects

There is growing evidence that ADT negatively affects men's psychosocial well-being (e.g., causing sexual dysfunction, bodily feminization) and physical health (e.g., increasing the risk of osteoporosis and metabolic syndrome). Although strategies for managing the majority of side effects exist, it is not clear that patients are benefiting from this knowledge. 

A recent study showed that more than 70% of 79 newly prescribed ADT patients did not know that anemia, memory problems, loss of body hair, and depression can occur following treatment. Moreover, over 50% were unaware of significant potential side effects such as reduced muscle mass, osteoporosis, increased fracture risk, weight gain, genital shrinkage, and gynecomastia [65]. The lack of awareness of ADT side effects may partially explain why ADT currently results in significant decreases in the quality of life of patients and their partners. 

Increased recognition of the side effects has resulted in strategies to minimize complications associated with ADT. Improved efforts to educate patients about treatment side effects and coping strategies may result in improved psychosocial and physical health for CaP patients undergoing ADT. Attempts to reduce ADT adverse effects include intermittent hormonal therapy and methods to reduce amount of intracellular androgens without reducing the circulating testosterone levels.

Due to the adverse events associated with ADT, the option of intermittent ADT therapy has been evaluated as a measure to reduce morbidity of treatment. It is reasonable to assume that both the acute and chronic complications of LHRH agonists would be ameliorated by delivering therapy in an intermittent mode. Prostate cancer is amenable to control by intermittent androgen suppression, affording these patients improved quality of life during time of therapy, with reduced toxicity and costs [66]. In a recent study, return of potency and resolution of anemia have been achieved with intermittent ADT [67]. Still, the unresolved issue is whether prostate cancer survival is negatively impacted by intermittent therapy. Currently, there are ongoing trials directly comparing continuous with intermittent hormonal therapy regimens. One of these trials is comparing intermittent ADT with continuous ADT in men with newly diagnosed metastatic prostate cancer [68]. With regard to side effects, there is evidence that intermittent ADT improves early side effects such as hot flashes, sexual activity, and fatigue, although its effect on long-term side effects remains inconclusive [44].
